# Barley-Water a Powerful Diuretic

**Published:** 1845-12

**Authors:** 


*Barley-water a powerful Diuretic.*—According to M. Themont, barley-water is a powerful diuretic, and may be employed with advantage in cases where more powerful diuretics might prove deleterious. He boils two handfuls of barley in three pints of water down to two pints, and gives a cupful five or six times daily. He relates a case of dropsy depending on hypertrophy of the heart, in which, by this simple means alone, a copious diuresis was produced which removed the dropsical swellings in a few days.—*Journ. de Farm.*

